# Supplementary material for: Automated assembly scaffolding using RagTag elevates a new tomato system for high-throughput genome editing
Source: Genome Biol. 2022 Dec 15;23:258. doi: 10.1186/s13059-022-02823-7 (PMC9753292; doi:10.1186/s13059-022-02823-7)
Supplement: Supplementary file 1 — Additional file 1. Supplementary Figures for Automated Assembly Scaffolding using RagTag elevates a new tomato system for high-throughput genome editing. [file 13059_2022_2823_MOESM1_ESM.pdf]

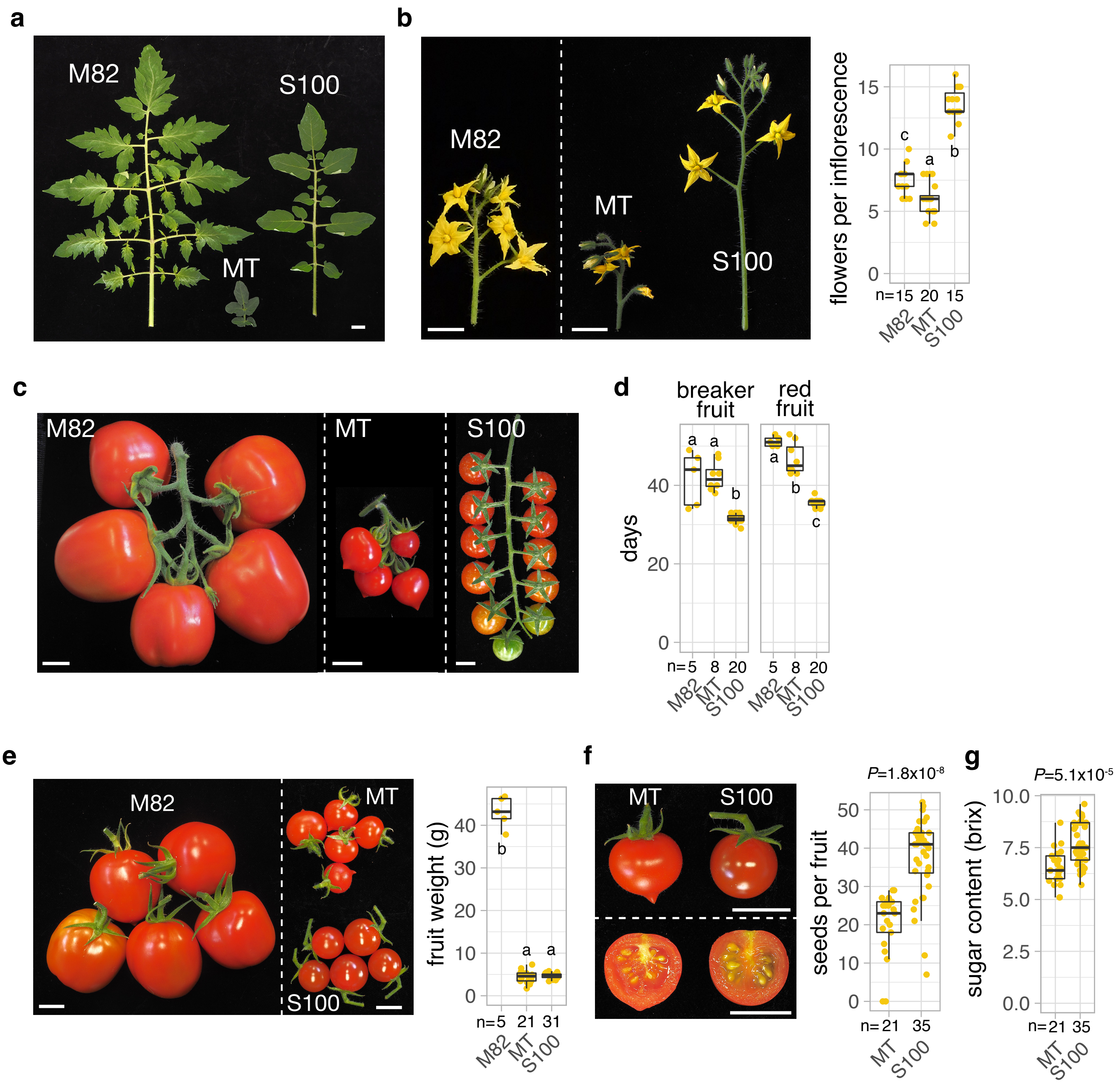

Fig. S1 | Phenotypic characteristics of the tomato cultivars M82 and Sweet-100 (S100), and the experimental variety Micro-tom (MT). a, Images of mature leaves from M82, MT, and S100. **b**, Images of detached inflorescences from M82, MT, and S100 plants and quantification of flower number per inflorescence. *n* equals the number of inflorescences. **c,** Images of detached fruit clusters from M82, S100 and MT. **d**, Quantification of days from anthesis to breaker fruit (left) and red fruit (right panel) in M82, MT, and S100. **e,** Images of fruits from M82, MT, and S100 and quantification of fruit weight. **f**, Images of fruits and quantification of seed number per fruit in MT and S100. n equals the number of fruits. **g**, Quantification of fruit sugar content in MT and S100. *n* in (d) to (g) equals the number of fruits. Scale bars indicate 1 cm. Letters in (b), (d), and (e) represent post-hoc Tukey's HSD tests. *P* values in (f) and (g) represent two-tailed, two-sample *t*-tests.

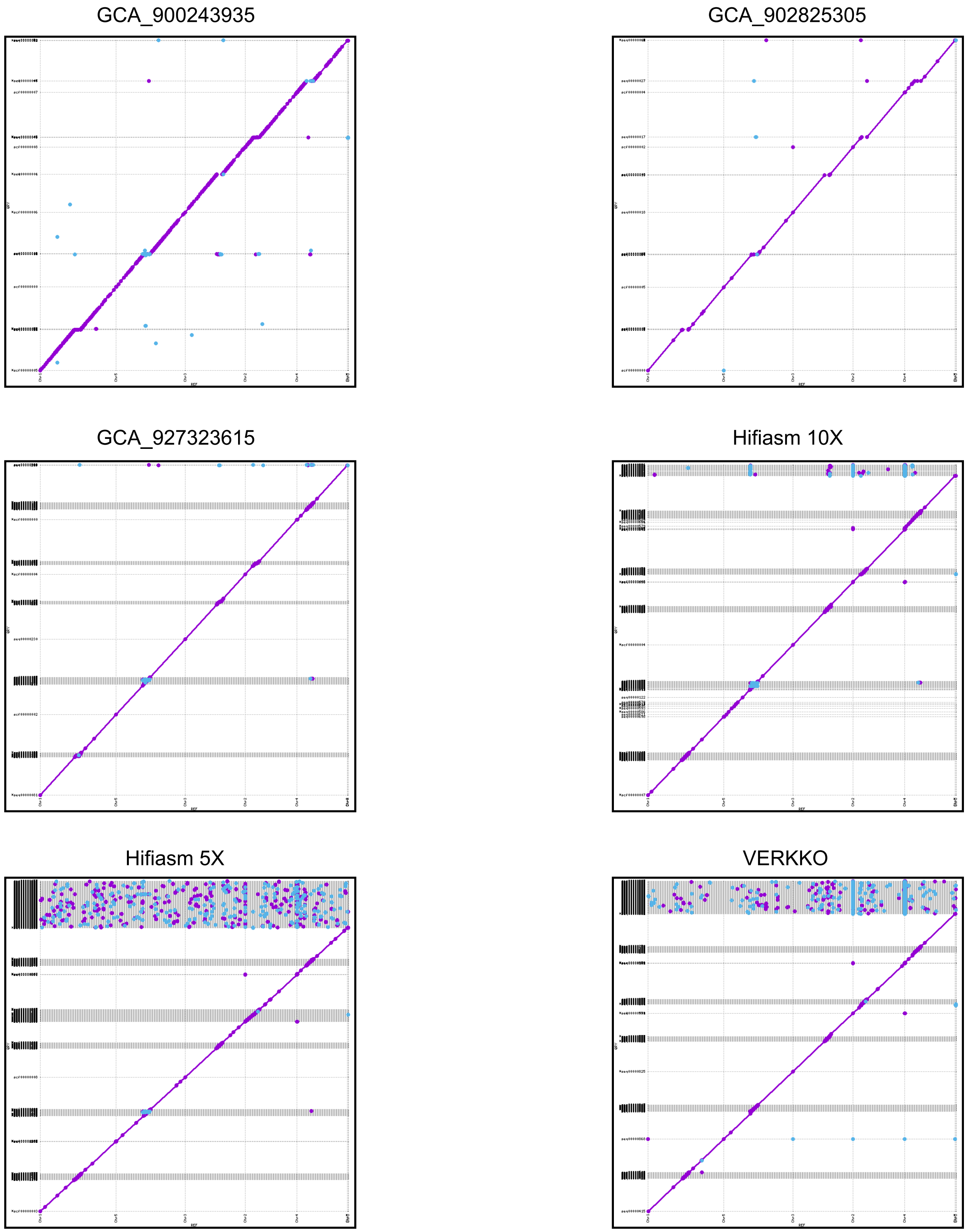

Fig. S 2 | Patching different *A. thaliana* assemblies with RagTag "patch". Dotplots comparing patched *A. thaliana* assemblies (y-axis) to the Col-CEN reference genome (x-axis). Purple and blue alignments indicate the forward and reverse strands, respectively.

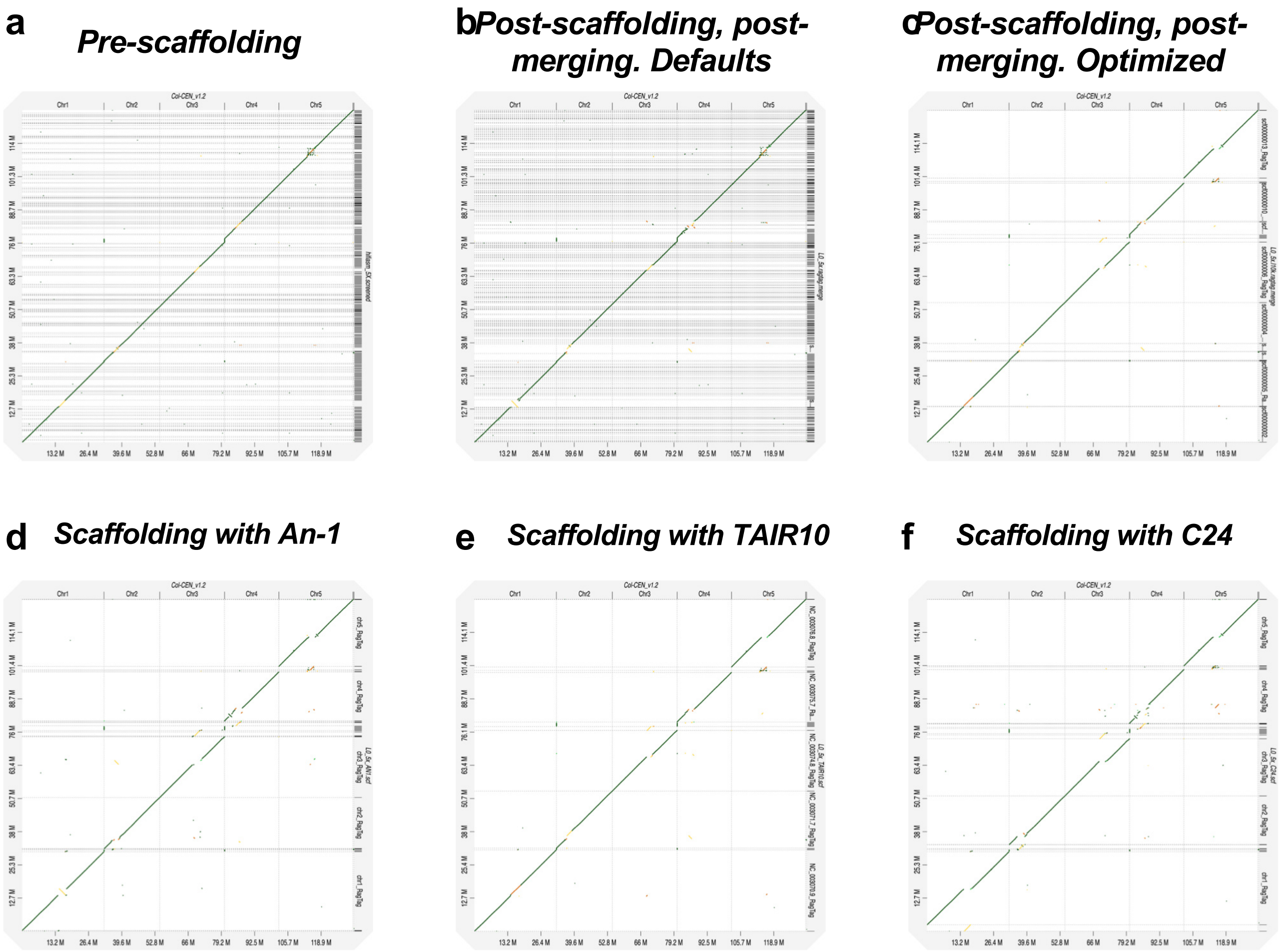

Fig. S3 | Merging Hifiasm assembly made with *A. thaliana* HiFi reads downsampled to 5x coverage with default and optimized RagTag "merge" parameters. Dotplots showing assemblies (y-axis) aligned to Col-CEN reference (x-axis). **a**, Pre-scaffolding, pre-merging assembly aligned to Col-CEN reference. **b**, Post-scaffolding and post-merging assembly aligned to Col-CEN reference, merged with default parameters (-l 100000). **c**, Post-scaffolding and post-merging assembly aligned to Col-CEN reference, merged with optimized parameters (-l 10000). **d**, Assembly scaffolded with An-1 reference and aligned to Col-CEN reference. **e**, Assembly scaffolded with TAIR10 reference and aligned to Col-CEN reference. **f**, Assembly scaffolded with C24 reference and aligned to Col-CEN reference.

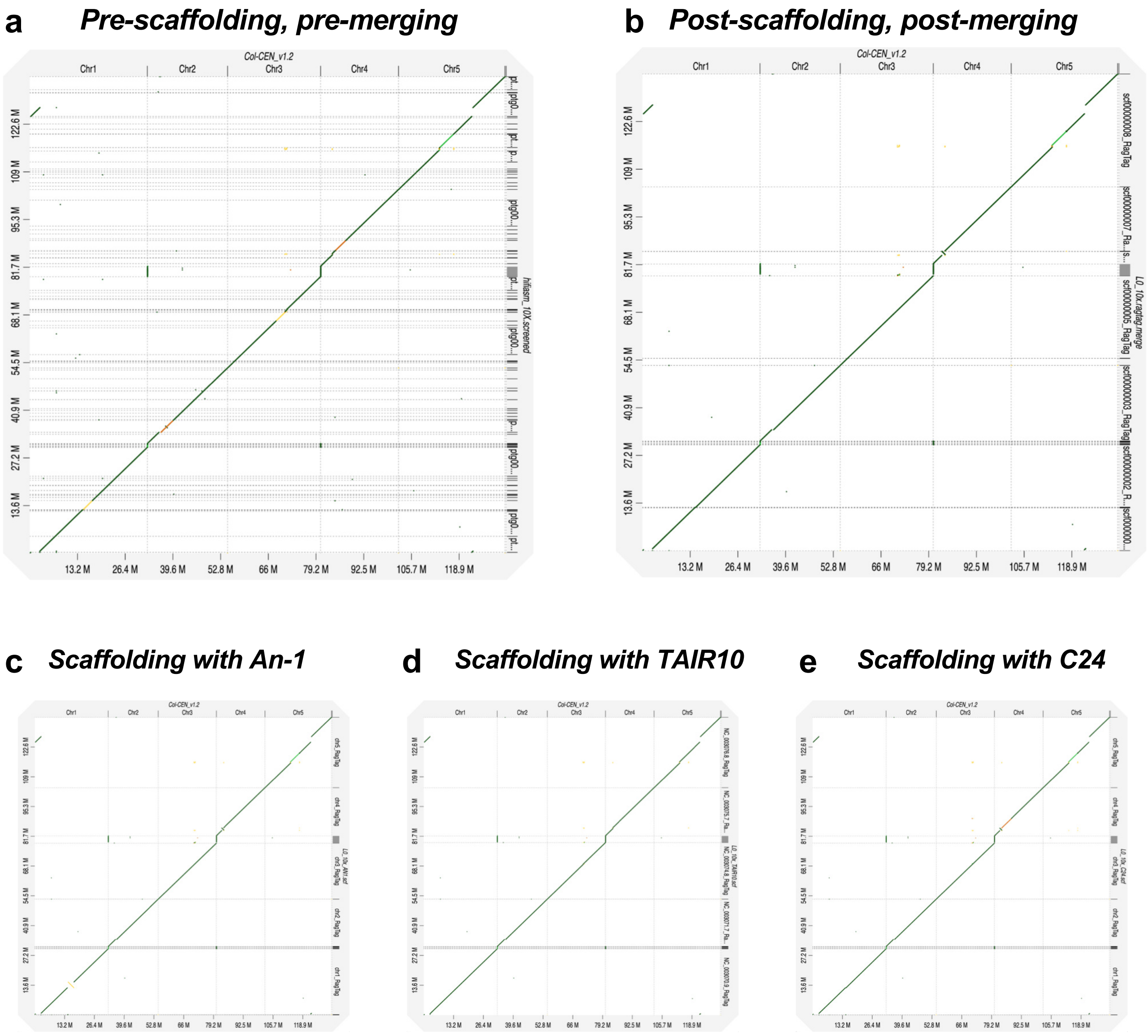

Fig. S4 | Merging Hifiasm assembly made with *A. thaliana* HiFi reads downsampled to 10x coverage with default RagTag "merge" parameters. Dotplots showing assemblies (y-axis) aligned to Col-CEN reference (x-axis). **a**, Pre-scaffolding, pre-merging assembly aligned to Col-CEN reference. **b**, Post-scaffolding and post-merging assembly aligned to Col-CEN reference. **c**, Assembly scaffolded with An-1 reference and aligned to Col-CEN reference. **d**, Assembly scaffolded with TAIR10 reference and aligned to Col-CEN reference. **e**, Assembly scaffolded with C24 reference and aligned to Col-CEN reference.

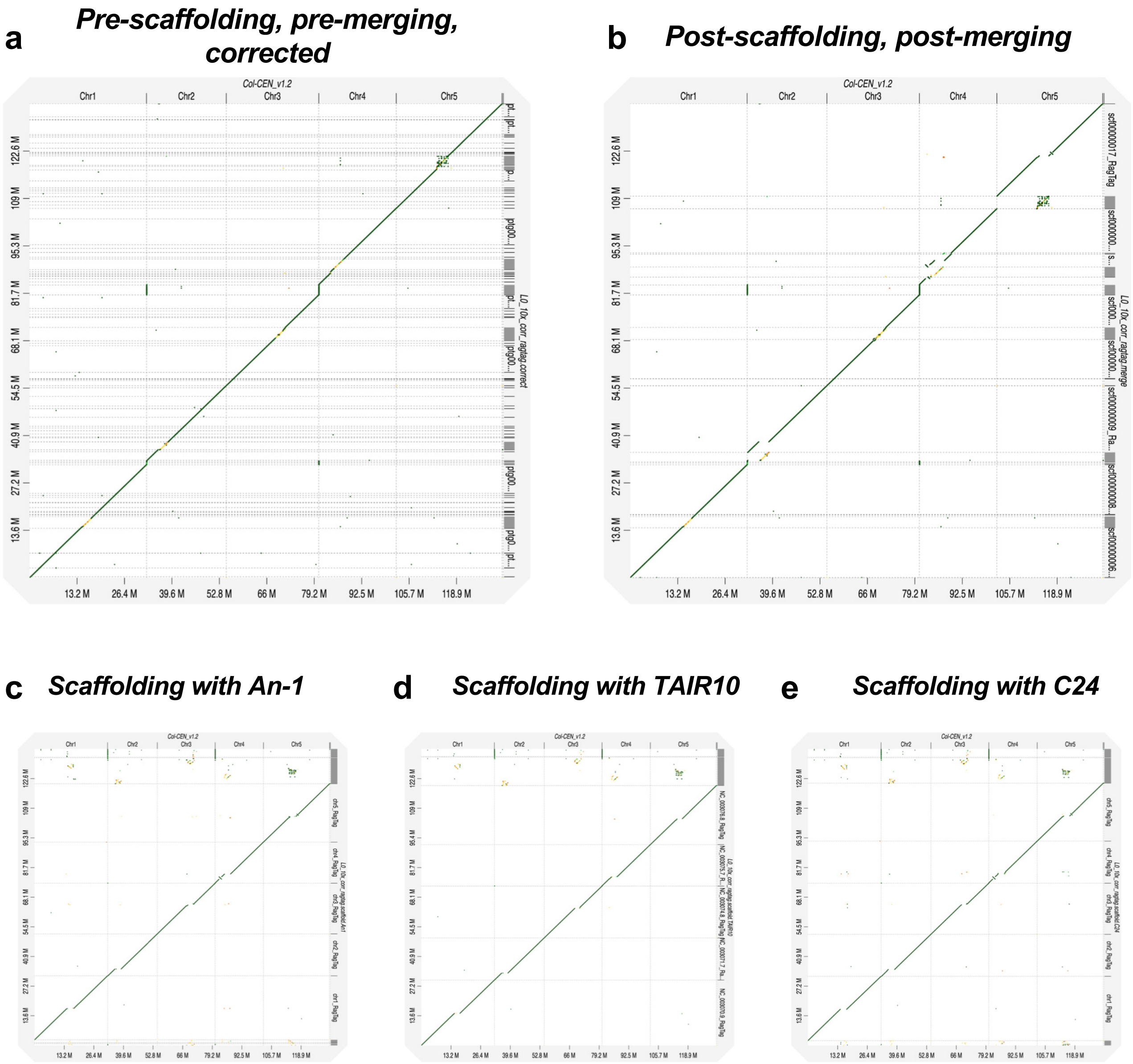

Fig. S5 | Merging corrected Hifiasm assembly made with *A. thaliana* HiFi reads downsampled to 10x coverage with default RagTag "merge" parameters. The resulting assembly was corrected with RagTag "correct" against the An-1 reference then merged with default RagTag "merge" parameters. Dotplots showing assemblies (y-axis) aligned to Col-CEN reference (x-axis). **a**, Pre-scaffolding, pre-merging assembly aligned to Col-CEN reference. **b**, Post-scaffolding and post-merging assembly aligned to Col-CEN reference. **c**, Assembly scaffolded with An-1 reference and aligned to Col-CEN reference. **d**, Assembly scaffolded with TAIR10 reference and aligned to Col-CEN reference. **e**, Assembly scaffolded with C24 reference and aligned to Col-CEN reference.

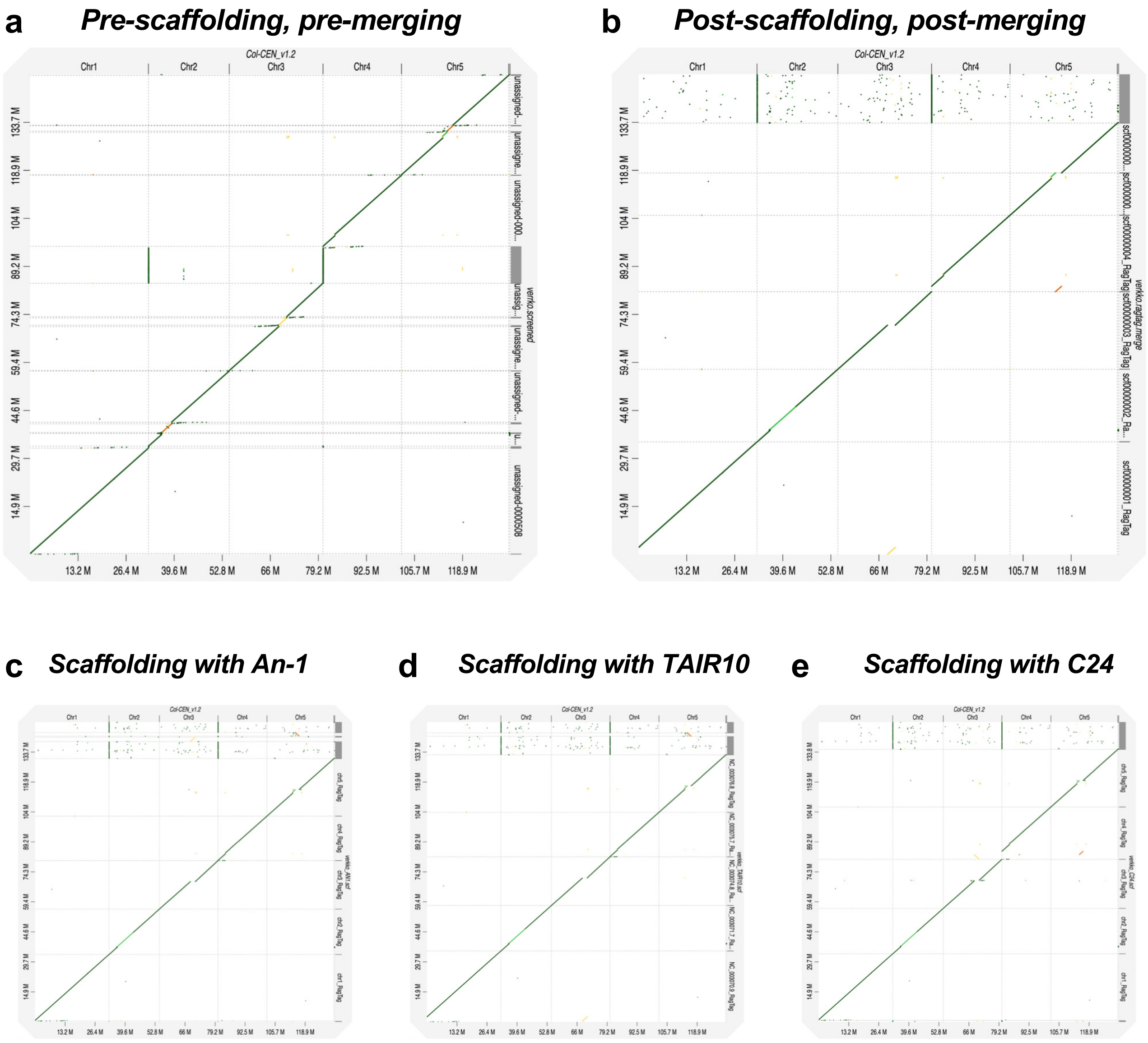

Fig. S6 | Merging Verkko assembly with default RagTag "merge" parameters. Dotplots showing assemblies (y-axis) aligned to Col-CEN reference (x-axis). **a**, Pre-scaffolding, pre-merging assembly aligned to Col-CEN reference. **b**, Post-scaffolding and post-merging assembly aligned to Col-CEN reference. **c**, Assembly scaffolded with An-1 reference and aligned to Col-CEN reference. **d**, Assembly scaffolded with TAIR10 reference and aligned to Col-CEN reference. **e**, Assembly scaffolded with C24 reference and aligned to Col-CEN reference.

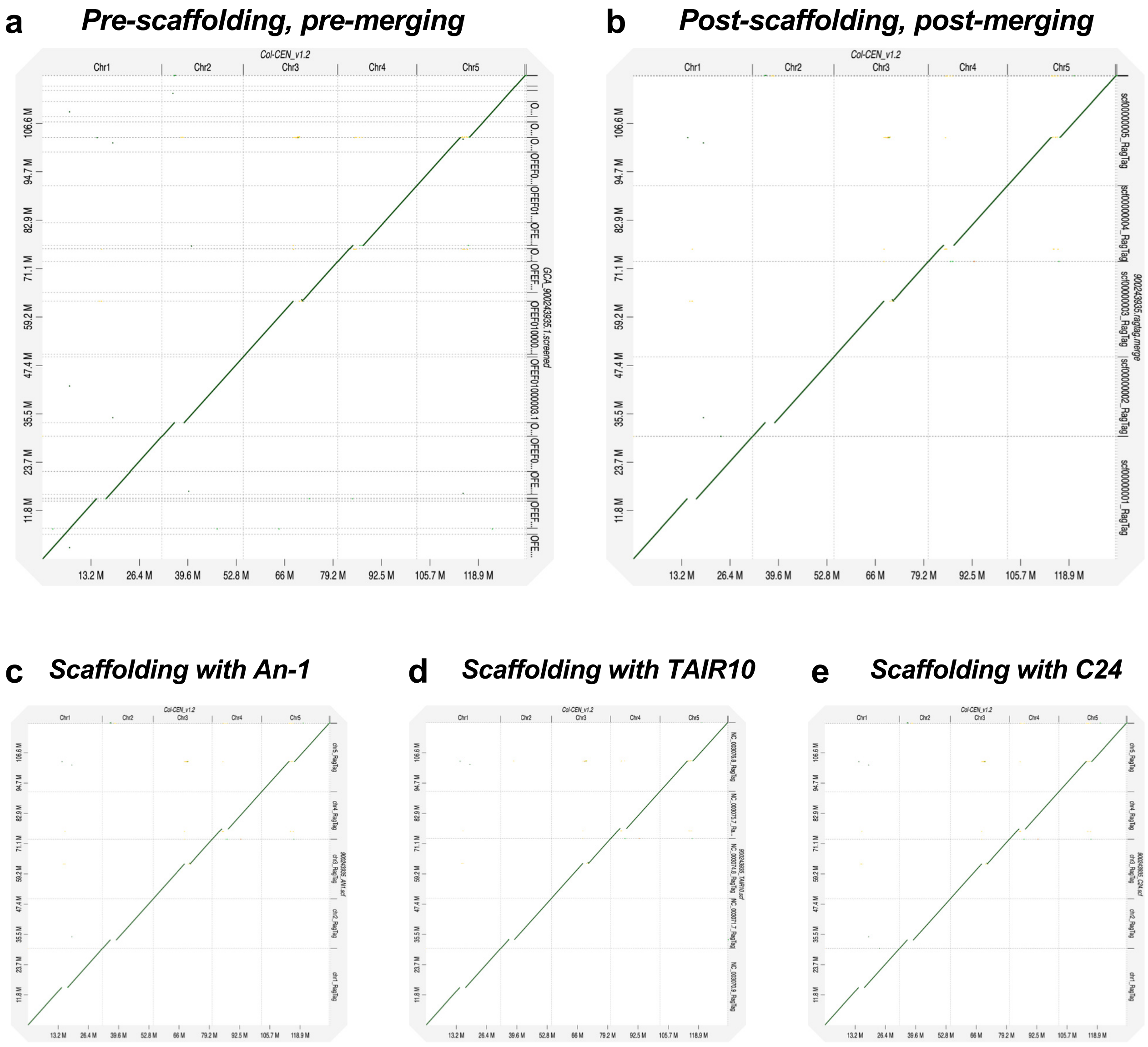

Fig. S7 | Merging 900243935 assembly with default RagTag "merge" parameters. Dotplots showing assemblies (y-axis) aligned to Col-CEN reference (x-axis). **a**, Pre-scaffolding, pre-merging assembly aligned to Col-CEN reference. **b**, Post-scaffolding and post-merging assembly aligned to Col-CEN reference. **c**, Assembly scaffolded with An-1 reference and aligned to Col-CEN reference. **d**, Assembly scaffolded with TAIR10 reference and aligned to Col-CEN reference. **e**, Assembly scaffolded with C24 reference and aligned to Col-CEN reference.

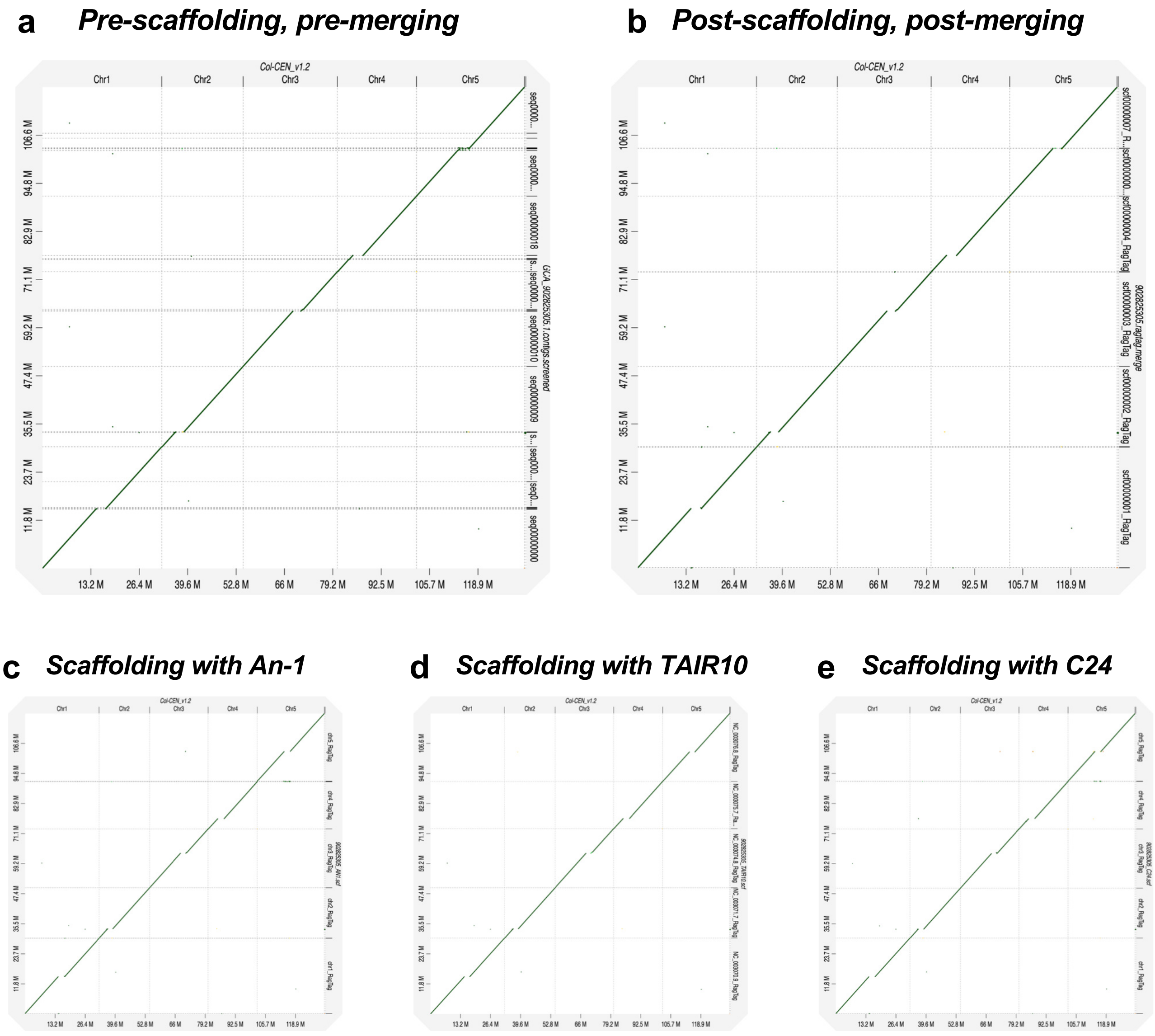

Fig. S8 | Merging 902825305 assembly with default RagTag "merge" parameters. Dotplots showing assemblies (y-axis) aligned to Col-CEN reference (x-axis). **a**, Pre-scaffolding, pre-merging assembly aligned to Col-CEN reference. **b**, Post-scaffolding and post-merging assembly aligned to Col-CEN reference. **c**, Assembly scaffolded with An-1 reference and aligned to Col-CEN reference. **d**, Assembly scaffolded with TAIR10 reference and aligned to Col-CEN reference. **e**, Assembly scaffolded with C24 reference and aligned to Col-CEN reference.

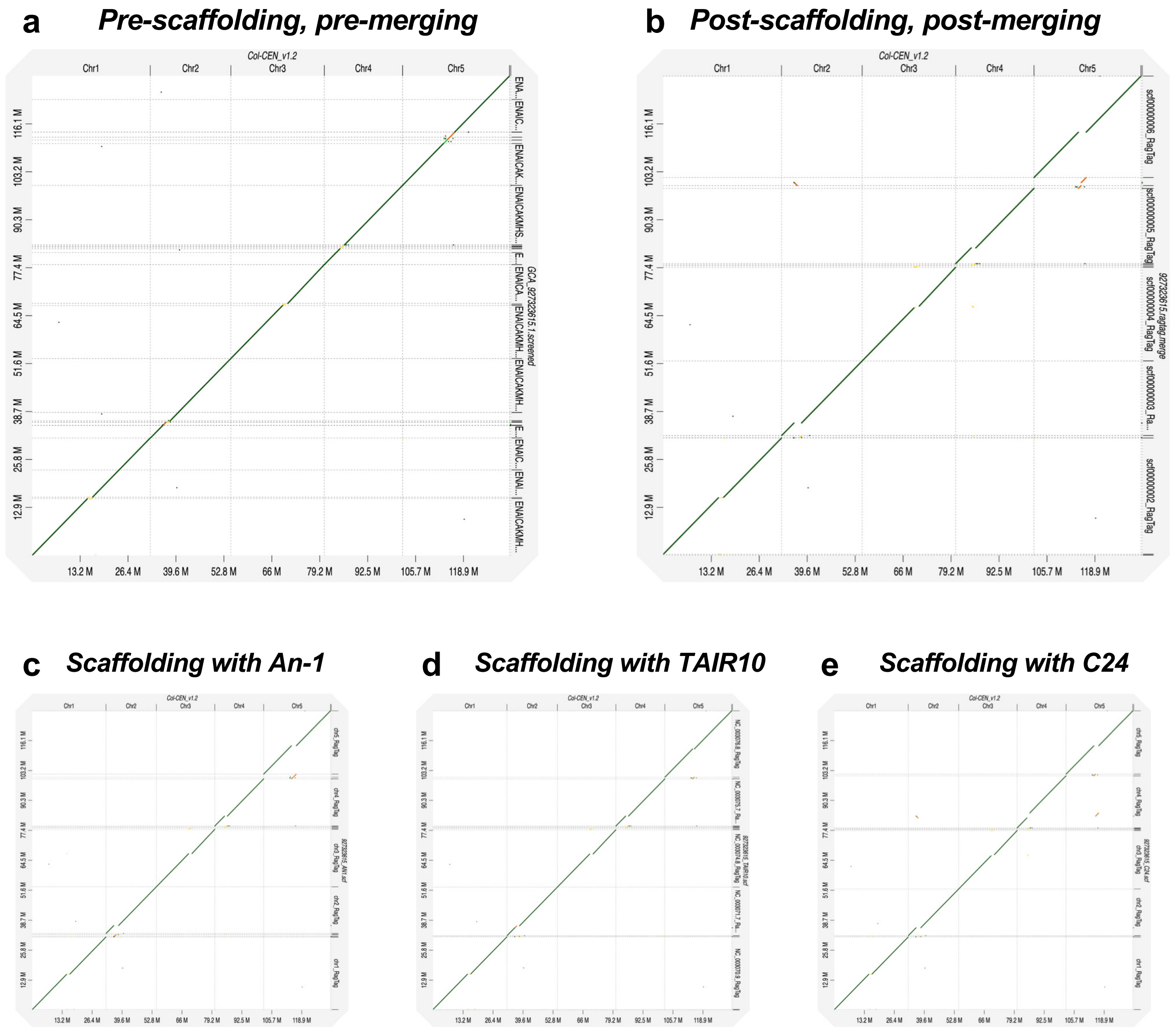

Fig. S9 | Merging 927323615 assembly with default RagTag "merge" parameters. Dotplots showing assemblies (y-axis) aligned to Col-CEN reference (x-axis). **a**, Pre-scaffolding, pre-merging assembly aligned to Col-CEN reference. **b**, Post-scaffolding and post-merging assembly aligned to Col-CEN reference. **c**, Assembly scaffolded with An-1 reference and aligned to Col-CEN reference. **d**, Assembly scaffolded with TAIR10 reference and aligned to Col-CEN reference. **e**, Assembly scaffolded with C24 reference and aligned to Col-CEN reference.

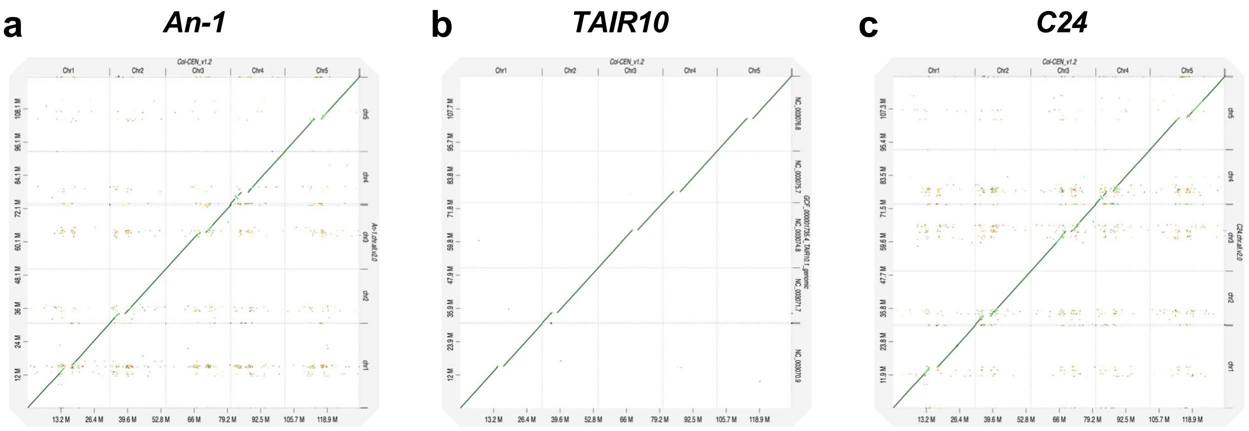

Fig. S10 | Reference assemblies used for scaffolding aligned to Col-CEN reference. **a**, An-1 reference (y-axis) aligned to Col-CEN (x-axis). **b**, TAIR10 (y-axis) aligned to Col-CEN (x-axis). **c**, C24 (y-axis) aligned to Col-CEN (x-axis).

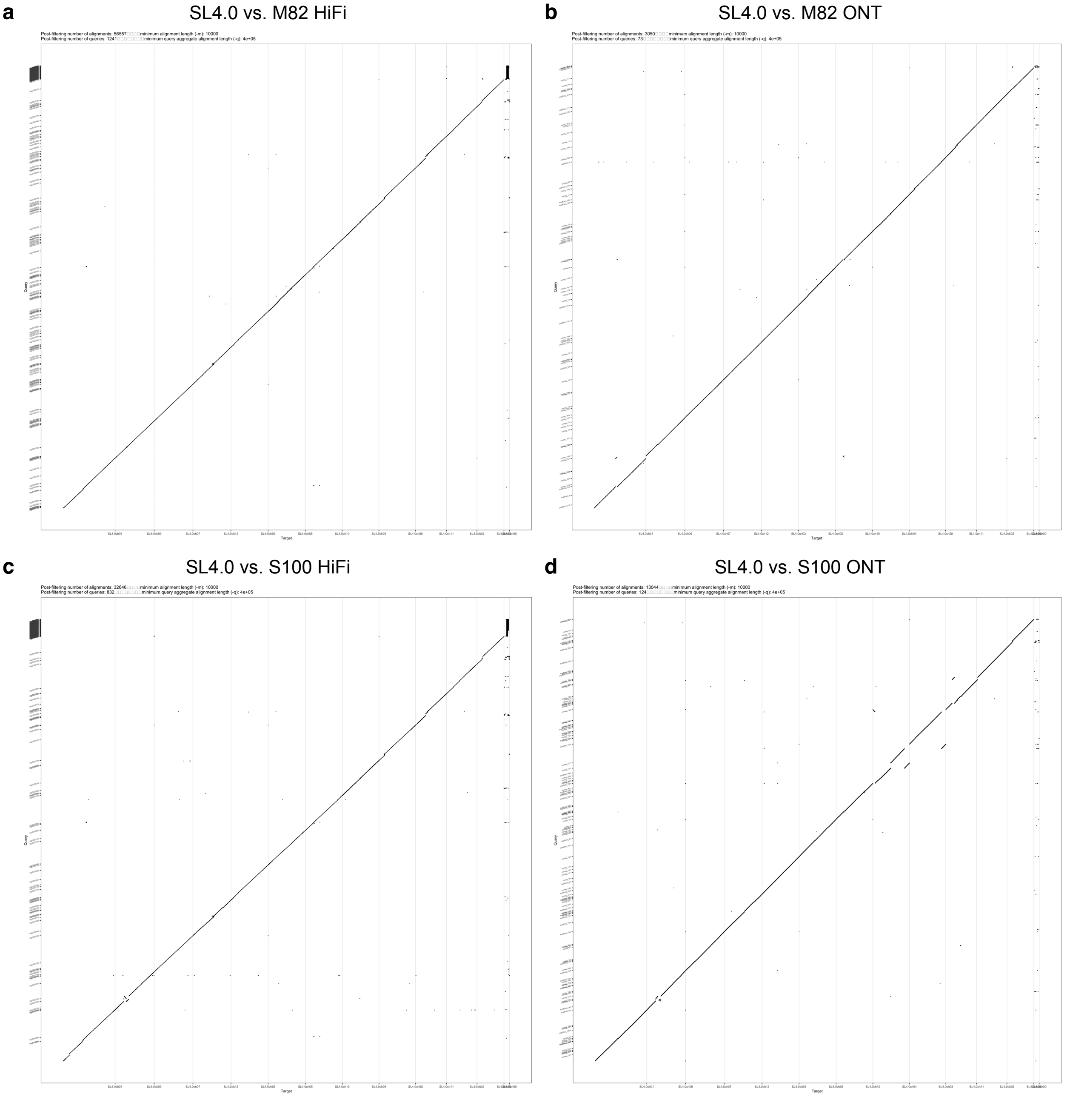

Fig. S11 | Dotplots comparing M82 and S100 draft assemblies to the tomato SL4.0 reference. a and b, Dotplots of M82 HiFi (a) and ONT (b) assemblies versus the tomato SL4.0 reference assembly. **c and d**, Dotplots of Sweet-100 HiFi (c) and ONT (d) assemblies versus the tomato SL4.0 reference assembly.

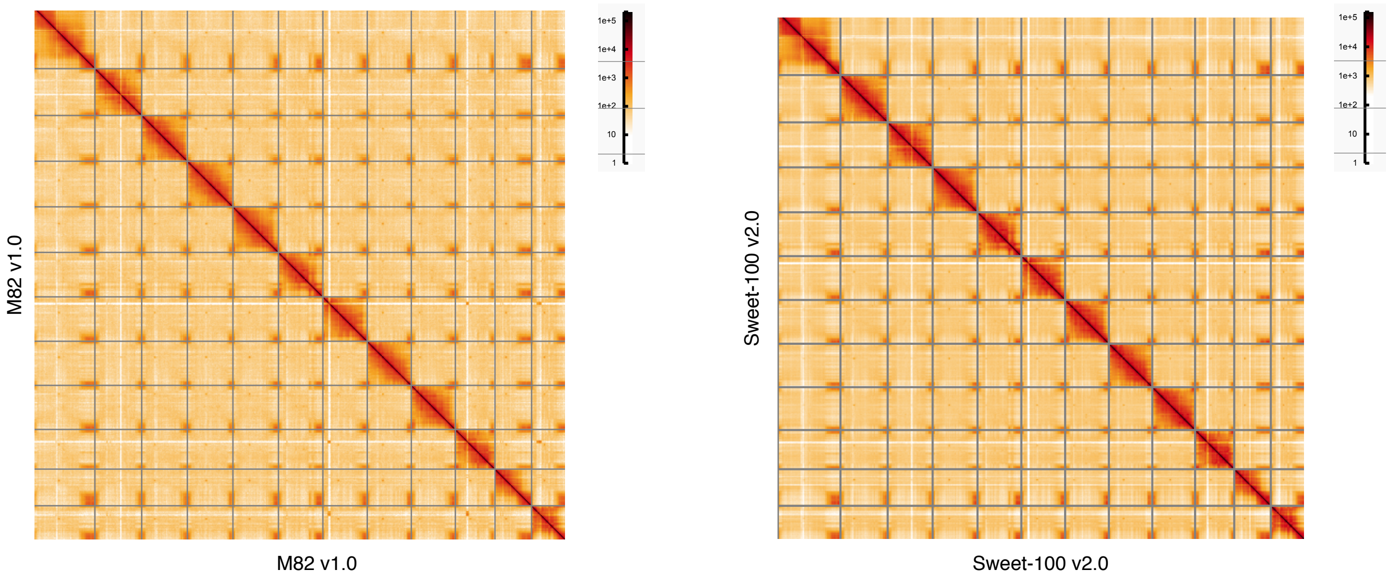

Fig. S12 | Hi-C heatmaps confirm reference assembly structural accuracy. Hi-C heatmaps for the M82 v1.0 and the Sweet-100 v2.0 reference assemblies. The 12 chromosomes are sorted from largest (top left) to smallest (bottom right).

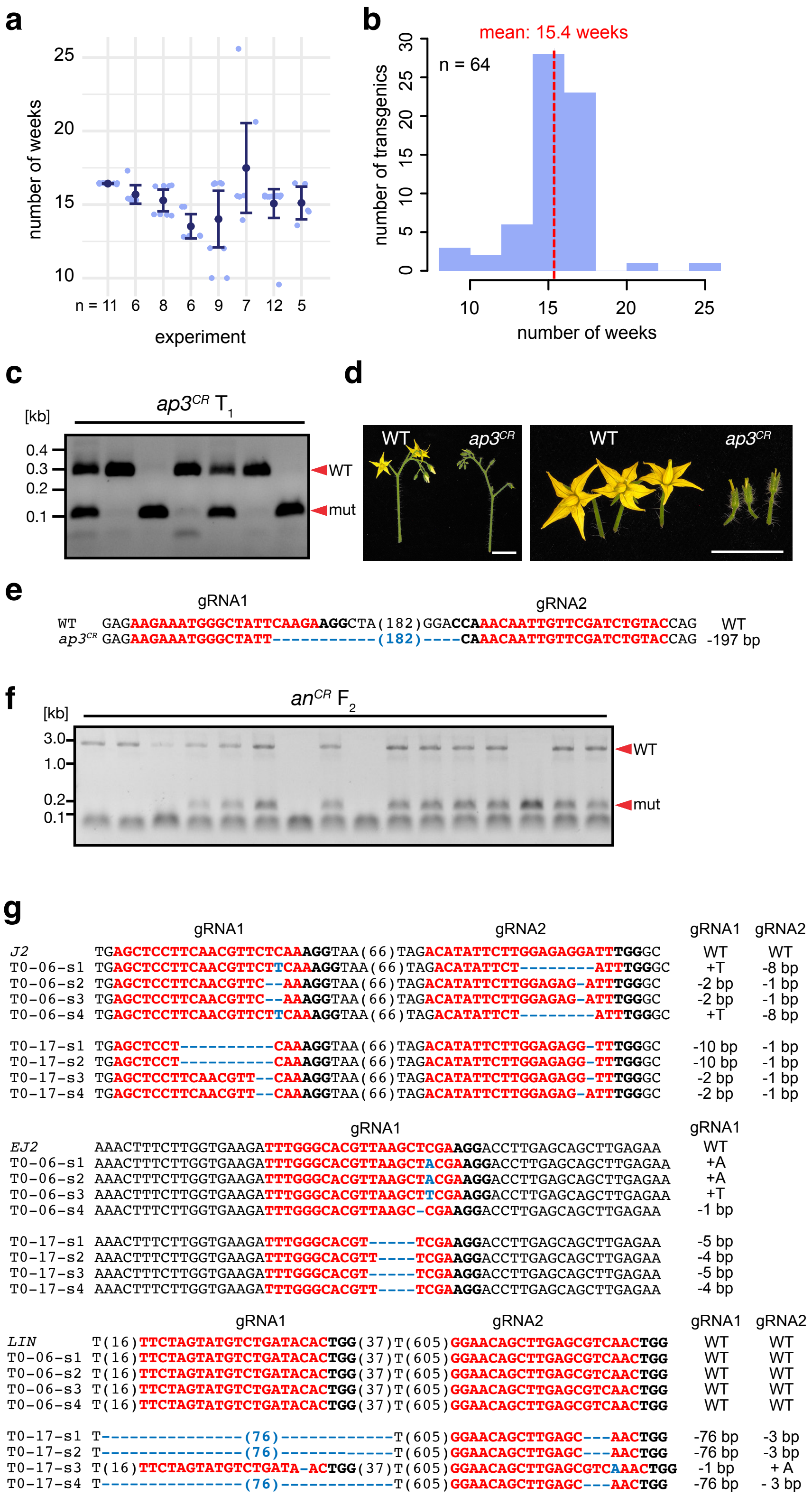

Fig. S13 | Plant transformation and CRISPR-Cas9 genome editing in Sweet-100. a and b, Number of weeks for obtaining transgenic plants, determined in weeks from sowing to transfer to soil. Data is shown for eight independent transformation experiments. n equals number of transgenic plants. **c**, PCR-genotyping for deletions in *SlAP3* in the non-transgenic second (F2) generation. **d**, Images of detached inflorescences (left) and flowers (right) from wild-type (WT) and *ap3CR* F2 plants. **e**, Sequence of CRISPR-induced *ap3CR* allele transmitted to *ap3CR* F2 plants. gRNA and PAM sequences are indicated in red and black bold letters, respectively; deletions are indicated with blue dashes; deletions; sequence gap length is given in parenthesis. **f**, PCR-genotyping for deletions in *AN* in the non-transgenic second (F2) generation. **g**, CRISPR-induced mutations in *J2*, *EJ2*, and *LIN* identified by Sanger sequencing of T0 plants. Scale bars indicate 1cm.
