# Supplementary material for: Automated assembly scaffolding using RagTag elevates a new tomato system for high-throughput genome editing
Source: Genome Biol. 2022 Dec 15;23:258. doi: 10.1186/s13059-022-02823-7 (PMC9753292; doi:10.1186/s13059-022-02823-7)
Supplement: Supplementary file 6 — Additional file 6. Review history. [file 13059_2022_2823_MOESM6_ESM.docx]

Review History

**First round of review**

**Reviewer 1**

**Are you able to assess all statistics in the manuscript, including the appropriateness of statistical tests used?** There are no statistics in the manuscript.

**Comments to author:**

The authors described RagTag, an update of RaGOO, with two new features (patch and merge) to allow for automatic scaffolding of genome assemblies. Using RagTag, the authors generated chromosome-level assemblies for the M82 and S100 genotypes of tomato. Finally, three sets of CRISPR-Cas9 experiments were presented suggesting the usefulness of the S100 genotype as an experimental system.
The new patching and merging algorithm are of potential use however the manuscript has limited results/benchmarks on their applicability (see comments below).
The text is well written and is mostly error free. However, the results and discussion sections are merged and making it a bit difficult to differentiate between the new results presented here and results from earlier work.
Major comments:
1) The examples for patching included in the manuscript are for cases when both HiFi and Nanopore reads were available. However, automated assembly is most useful when working with many genome assemblies. In such cases, generating both HiFi and Nanopore reads for each to be assembled genome might not be feasible. Is it possible to use patching algorithm when only one type of reads/assemblies are available? Or would that only be the merging?
Additionally, benchmarks are required on the effect of quality/continuity of the initial contig assemblies on the patching and merging algorithms?
2) The two examples (tomato and CHM13) are both effectively haploid genomes. The scaffolding performance on heterozygous genomes is not clear. What about polyploids? If these can be scaffolded as well, then more results are required otherwise these limitations should be discussed.
3) The introduction lacks a description of the currently available methods for genome patching and merging (for example: https://academic.oup.com/g3journal/article/6/7/1899/6027724 , https://genomebiology.biomedcentral.com/articles/10.1186/s13059-017-1213-3) and the improvements made in RagTag. As there are quite some methods that are already available for genome assembly reconciliation, performance comparison of the merging algorithms are required.
4) For the two tomato genomes assembled here, merging steps used the homology-based scaffolds and Hi-C based scaffolds. However, it is not clear what were the improvements of the merge step comparison to initials scaffolds. It is also not clear how the merged assembly quality is affected by the number of scaffolded assemblies that are being merged. Also, does the type of scaffolding (homology-based, Optimal Maps, HiC etc) affect the merging?
5) In Supplementary Fig 2, (from left to right) chromosome 1, 6, 11, 12 for M82 and chromosome 1,5,11,12 for Sweet 100 have HiC signals typical for mis-assemblies. What is the cause for this? Also I find the text (line 25-27, page 4) to be inconsistent with these results and more accurate phrasing would be better.
6) It is unclear to me what does "elevated a rapid-cycling tomato variety...editing experiments" in the conclusion mean and how it is supported by the results. The authors presented the S100 system earlier (https://www.nature.com/articles/ng.3733) and so are the capabilities of doing CRISPR/Cas9 with it (https://www.cell.com/cell/fulltext/S0092-8674(17)30486-5). Here the authors present the genome assembly for S100. Are there other improvements that have been made in the system as well? If the assembly is the only improvement, then it should be made clear how the automated assembly with RagTag improves the usability of S100 in contrast to an assembly from current methods with more manual curation.

Minor Comments:
1) At page 3: line 25, would not Soyk et al. Nat Gen 2016 be a more suitable citation?
2) Page 4: line 36, typo in fig reference.
3) Page 5: line 6, in ap3CR it is not clear what is CR.
4) Page 6: line 23, function h() is referred but I could not find it in Methods.
5) Related to 4), would be better if reference to Method sections also include name of the sub-sections.
6) Supp Fig 1, do not have 'n' as mentioned in the caption.
7) The sequencing data is available on SRA, the assemblies should also be uploaded to standard public servers (NCBI, ENA etc).

**Reviewer 2**

**Are you able to assess all statistics in the manuscript, including the appropriateness of statistical tests used?** Yes, and I have assessed the statistics in my report.

**Comments to author:**

This manuscript describes the assembly of high-quality chromosome-scale genomes of two tomato cultivated accessions (M82 and Sweet-100) using their newly developed (or improved) package RagTag and the promotion of a rapid-cycling and compact growth variety (Sweet-100) as an effective system for tomato functional genomics and genome editing studies. Overall, the manuscript is well-written and the resources and computational programs developed by the authors are very useful and valuable.

Page 3, Line 43, "RagTag, a new toolset": Since RagTag seems to be a newer version of RaGOO, maybe change this to "RagTag, an improved version of RaGOO with several newly added functions"?

Page 3, Line 55: "[12]. [12]" -> "[12]."

Page 4, Line 36: "Fig. 1j,kf,g" -> "Fig. 1j,k"

Page 11, Line 30-49: I don't think this paragraph fits well to this manuscript. I would suggest to move this paragraph to the RagTag website.

Page 13, Line 31: Wrong citation for Winnowmap2. Ref. 23 is for Minimap2. Should be https://www.biorxiv.org/content/10.1101/2020.11.01.363887v1

Page 13, Line 48 and other places: The authors mentioned using Hi-C data to help scaffold contigs of both Sweet-100 and M82. Are the data generated by the authors under this study? I couldn't find the description of Hi-C data generation. Or the data were downloaded from public depositories? Need to make this clear.

Page 14, Line 14-17: Why the tomato chloroplast and mitochondrion genomes from GenBank were added to final assembly? These should be independent of nuclear genome assemblies.

Page 14, Line 24: Ref. 35 should be cited for Sniffles. Remove Ref. 35 in Line 27.

Page 15, Line 11: Change "Mercury" to "Merqury". Need to indicate what data were used for Merqury analyses. Illumina short reads or HiFi long reads…

**Reviewer 3**

**Are you able to assess all statistics in the manuscript, including the appropriateness of statistical tests used?** There are no statistics in the manuscript.

**Comments to author:**

The manuscript is introducing RagTag as a successor to Ragoo. Ragoo in itself was highly useful already and I think RagTag is a very useful contribution.

I guess dictated by the form(?) the manuscript presents RagTag - but not in the depth it deserves - and also genome editing results.

Like this I have a feeling the manuscript is neither fish nor fowl....

Considering this should be a toolpaper for RagTag:

- The individual assembly statistics (N50, 70 etc) for the Sweet-100 tomato assembly should be presented and at least some dot plots between the hifi and the ONP assembly be shown maybe referenced versus the reference tomato genome.

- The authors might want to investigate other assemblers for the ONP data Vanrengs reported better results for the -however seemingly unmaintained - necat assembler;

- Definitely a comparison to quickmerge needs to be included as this can be used for merging ONT + hifi data as well

- The authors should best provide this test case in the github and state what should be the best strategy for Patching hifi with ONP or vice versa? The strategy is hidden in the methods section here and it makes sense as hifi is more base-correct as discussed but for maximal usability a clear guideline should be given in the documentation.

-For the human genome I think this deserves a figure in the main manuscript as well.

-Can the error in the patched human genome be resolved by using a different ONP genome?

- Would there be any warnings in the the RagTag patch procedure so one could be aware of this problem

=>I am asking this question as I suspect but might be wrong that RagTag is going to be highly used for non model genomes and to investigate structural variation in human genomes where both technologies are available. Here it was easy to find such an issue (it is major and easy to spot) but what about lesser problems

-In the same vein maybe the authors should assembly non perfect human genome assemblies and test RagTag on these to investigate the potential performance in high throughput cancer genome analysis

The biological story could then be a nice add on story to show that RagTag is not only an interestig tool but that it has direct applications.

**Authors Response**

**Point-by-point responses to the reviewers’ comments:**

Reviewer 1 comment 0

The authors described RagTag, an update of RaGOO, with two new features (patch and merge) to allow for automatic scaffolding of genome assemblies. Using RagTag, the authors generated chromosome-level assemblies for the M82 and S100 genotypes of tomato. Finally, three sets of CRISPR-Cas9 experiments were presented suggesting the usefulness of the S100 genotype as an experimental system. The new patching and merging algorithm are of potential use however the manuscript has limited results/benchmarks on their applicability (see comments below). The text is well written and is mostly error free. However, the results and discussion sections are merged and making it a bit difficult to differentiate between the new results presented here and results from earlier work.

***Response:***

***We thank the Reviewer for the positive comments on the potential of the RagTag method and the usefulness of S100 genotype as an experimental system. We also appreciate the suggestions to improve the manuscript, including additional analyses for benchmarking the RagTag software. Below, please find our responses to all questions and comments.***

Reviewer 1 comment 1

The examples for patching included in the manuscript are for cases when both HiFi and Nanopore reads were available. However, automated assembly is most useful when working with many genome assemblies. In such cases, generating both HiFi and Nanopore reads for each to be assembled genome might not be feasible. Is it possible to use patching algorithm when only one type of reads/assemblies are available? Or would that only be the merging? Additionally, benchmarks are required on the effect of quality/continuity of the initial contig assemblies on the patching and merging algorithms?

***Response:***

***We thank the reviewer for raising these points. RagTag “patch” will work on any two assemblies. Certainly, a reasonable application would be to assemble one dataset with two different assemblers. Then, one could use one assembly to patch another. Another application is to patch a draft assembly with an existing reference assembly. On page 3, we now state that while utilizing complementary data types is ideal, RagTag is generic and can be used on any pair of assemblies.***

***To benchmark the impact of input assembly quality, we ran RagTag “patch” and “merge” on several Col-0 A. thaliana genome assemblies of varying quality. We then assessed the accuracy of the RagTag results by comparing output assemblies to the highly accurate/complete Col-0 “Col-CEN” assembly [1]. As expected, there is a correlation between input and output assembly quality. We now describe this analysis on page 3 and page 12 and we include the corresponding QUAST results in Supplementary Table 1,2 .***

***Text changes:***

***On page 3, we now write:***

***However, while utilizing complementary types of sequencing data is ideal, RagTag “patch” is generic and can be applied to any pair of assemblies.***

***RagTag can be used for assemblies of varying quality, though benchmarking with several Arabidopsis thaliana Columbia-0 draft genome assemblies indicated that higher quality input assemblies led to more accurate RagTag results (Supplementary Table 1,2).***

Reviewer 1 comment 2

The two examples (tomato and CHM13) are both effectively haploid genomes. The scaffolding performance on heterozygous genomes is not clear. What about polyploids? If these can be scaffolded as well, then more results are required otherwise these limitations should be discussed.

***Response:***

***We thank the reviewer for raising this point, especially because it is a very common question from RagTag users. We have provided additional documentation to the GitHub repository to provide more clarity for users:***

***https://github.com/malonge/RagTag/wiki/FAQ#1-can-ragtag-work-with-multiple-haplotypes-or-polyploids***

***In summary, RagTag will work on any genome assembly, provided that it is reasonably accurate and that it adheres to certain conventions. Specifically, it requires that assemblies should only represent one haplotype/homologous genome. In other words, at each locus, the assembly should only ever represent one haplotype/homologous allele. Every major database/consortium adheres to this convention. For example, when haplotype-resolved human genomes are published, the haploid genomes are published as two separate genome assemblies. Alternatively, non-haplotype resolved assemblies are commonly published as “pseudo-haploid” sequences where the two haplotypes are “squashed” into a single representation.***

***Performing experiments on heterozygous genomes would therefore be unremarkable because RagTag works on individual genome assemblies, and such assemblies (as described in the new documentation) should only ever represent one allele at each locus. The way this is achieved (e.g. “pseudohaploid” or “squashed” vs. “dual” vs. “haplotype-resolved”) will usually have little or no effect on RagTag results. Even if the effects were pronounced, that would be influenced by the chosen reference genome or the whole genome aligner, not RagTag. If users would like to work with many dual or haplotype-resolved assemblies, they can simply run RagTag once for each assembly. For example, for healthy diploid human genomes, users can run RagTag once for a “squashed” human genome assembly, or twice — once for each of two haplotype-resolved genome assemblies.***

***False duplication due to redundant haplotypes are a common type of misassembly. Since RagTag cannot correct these misassemblies and because they could conceivably cause errors in the RagTag output, we advise users to remove such misassemblies before using RagTag. This advice is now stated in the new documentation.***

***The above also pertains to diploids and autopolyploids. As for allopolyploids (where multiple homoeologous alleles will be represented in the same assembly), the subgenomes will often be divergent enough to effectively be treated as distinct chromosomes in genome assemblies [2]. Therefore, any new analysis with allopolyploids would also be unremarkable in this respect. This is demonstrated by our use of RaGOO/RagTag to scaffold an allohexaploid bread wheat genome (Chinese Spring) [3].***

Reviewer 1 comment 3

The introduction lacks a description of the currently available methods for genome patching and merging (for example: https://academic.oup.com/g3journal/article/6/7/1899/6027724, https://genomebiology.biomedcentral.com/articles/10.1186/s13059-017-1213-3) and the improvements made in RagTag. As there are quite some methods that are already available for genome assembly reconciliation, performance comparison of the merging algorithms are required.

***Response:***

***Thank the reviewer for these suggestions. On page 2, we now mention “genome assembly reconciliation” with corresponding citations.***

***For a performance comparison, we compared RagTag “patch” to Quickmerge, Dentist, TGS-GapCloser and SAMBA. TGS-GapCloser fills gaps but does not join contigs, therefore it didn’t make any improvements to our gapless input contigs. DENTIST failed to make any changes to the assemblies. We are confident that we ran the software correctly, but we have opened a GitHub issue to confirm our approach: https://github.com/a-ludi/dentist/issues/22***

***SAMBA and Quickmerge both produced updated results. In order to assess/compare the accuracy of RagTag, SAMBA, and Quickmerge, we compared these assemblies to the final published M82/S100 assemblies as well as to the SL4.0 reference with QUAST. This analysis did not indicate that either software was clearly superior in terms of accuracy. For example, the RagTag M82 assembly contained the fewest misassemblies when compared to M82v1.0 and SL4.0. S100 had the fewest misassemblies compared to S100v2.0 but not compared to SL4.0. These results suggest that RagTag “patch” is generally superior in terms of precision. However, since our analysis is not definitive, we only state that the tools are “competitive” with each other in the manuscript. We now describe this analysis on page 3 and the QUAST results are included in Supplementary Table 4.***

***We did not perform any comparisons for RagTag “merge” because we do not know of any tools that perform an analogous task. Many tools that use the term “merge” do what we describe as “patching”. Our use of the term “merge” specifically refers to the merging of scaffolding solutions (i.e. AGP files), whereas other tools seek to merge genome assemblies. In other words, RagTag seeks to merge/reconcile the contig ordering and orienting hypotheses while other tools seek to merge the actual contigs.***

***Text changes:***

***On page 2, we now write:***

***Combining complementary genome assemblies is sometimes referred to as “reconciliation” and several tools have been developed for this task [12,13].***

***On page 3, we now write***

***A comparison to other patching tools showed that RagTag “patch” outperformed DENTIST [18] and it was competitive with SAMBA [19] and Quickmerge [20] (Supplementary Table 4)***

Reviewer 1 comment 4

For the two tomato genomes assembled here, merging steps used the homology-based scaffolds and Hi-C based scaffolds. However, it is not clear what were the improvements of the merge step comparison to initials scaffolds. It is also not clear how the merged assembly quality is affected by the number of scaffolded assemblies that are being merged. Also, does the type of scaffolding (homology-based, Optimal Maps, HiC etc) affect the merging?

***Response:***

***We thank the reviewer for this comment. Unlike the homology-based scaffolds and the merged scaffolds, our M82 Hi-C-based scaffolds did not achieve full chromosome scale. We have included assembly stats for the homology-based and Hi-C-based scaffolds in Supplementary Table 3 to make this point more clear.***

***We have included a new analysis to demonstrate the differences between the scaffolded assemblies. We ran QUAST to compare the different scaffolded assemblies to the final packaged assemblies (M82 v1.0 and S100 v2.0) and report the results in a new Supplementary Table 5). For M82, we compared the homology-based, the Hi-C-based, and the merged scaffolds. For S100, we compared both homology-based scaffolds, as well as the Hi-C-based and the merged scaffolds. Of course, the merged assembly was used to generate the final packaged assembly, so the analysis is not an unbiased accuracy measurement. However, because the packaged assemblies were extensively QCed and ultimately approved via manual curation, we think it is useful to know how close the initial scaffolds were to the final product. While all S100 scaffolds are different from each other, the M82 merged and homology-based scaffolds are the same. However, even though RagTag “merge” outputs the same scaffolds as homology-based input scaffolds (and the S100 scaffolds are similar), the merge results have utility because they represent a validation of the homology-based scaffolds. Because the Hi-C scaffolds did not contradict the homology-based scaffolds, we could be more confident that there were no scaffolding errors in the homology-based scaffolds due to reference bias (later confirmed by manual validation). In other words, the utility of RagTag “merge” is to validate the input scaffolds and/or to synergistically improve them, a point that we now make clear in the results. We now refer to these new QUAST results in the results section.***

***RagTag “merge” reconciles multiple scaffolding solutions. In practice, as the number of solutions grows, small differences between scaffolding solutions (due to a number of factors including errors and true genetic differences between genotypes/haplotypes) can “add up” and induce scaffolding errors. Therefore, we recommend that users only use a minimal number of informative/complementary maps. For example, if users have Hi-C-based, Optical map-based, and homology-based maps, it is reasonable to include one scaffolding solution per map type. However we would not recommend using multiple scaffolding solutions per map type (e.g. from different scaffolding algorithms). Rather, one should use the single best scaffolding solution for each map. We have added text to the methods to highlight this point***

***RagTag merge is generic and it does not treat maps of different technologies differently. Results should only depend on the accuracy of the input scaffolding solutions, which of course can correlate with map technology.***

***Text changes:***

***On page 3, we now write:***

***We incorporated a variety of physical and spatial proximity maps into RagTag “merge” to build 12 chromosome-scale pseudomolecules for each assembly (Supplementary Table 5). For S100, RagTag synergistically combined three distinct scaffolding solutions (two homology-based and one Hi-C based) to generate a new solution. While two distinct scaffolding solutions were used as input for M82 (homology-based and Hi-C-based), the output was identical to the homology-based input scaffolds. This agreement between the homology-based and the Hi-C-based scaffolds served as a validation of the homology-based scaffolds.***

Reviewer 1 comment 5

In Supplementary Fig 2, (from left to right) chromosome 1, 6, 11, 12 for M82 and chromosome 1,5,11,12 for Sweet 100 have HiC signals typical for mis-assemblies. What is the cause for this? Also I find the text (line 25-27, page 4) to be inconsistent with these results and more accurate phrasing would be better.

***Response:***

***We thank the reviewer for this comment. We certainly want to ensure that our assemblies are structural accurate and that any notable misassembly will be corrected. Prior to the first submission, we manually validated every scaffolding join in Juicebox assembly tools. We also used SV callers (described in the methods) to detect potentially misassemblies and/or heterozygous SVs. We have reviewed the contact maps again and we believe that, as we stated in the manuscript, the assemblies are structurally accurate and that Hi-C supports this claim. We believe that you may be referencing genuine 3D contact signals rather than signals of misassemblies.***

***To better demonstrate this, we provide zoomed-in contact maps for these chromosomes (figures are presented in the same order that you specified above). We have also made the “.mcool” files available for download for you to browse the data in HiGlass. If you still disagree, we hope to have the opportunity to either correct the misassembly or demonstrate the accuracy of the current assembly.***

***Download link for M82 .mcool file:***

***https://drive.google.com/drive/folders/1EDu1JDzXzdZeYeyMM4f6s0ST06J_Egqr?usp=sharing***

***Download link for S100 .mcool file:***

***https://drive.google.com/drive/folders/1RH06hoS815cajBnDUZAvlCS2VwUsUAl6?usp=sharing***

Reviewer 1 comment 6

It is unclear to me what does "elevated a rapid-cycling tomato variety...editing experiments" in the conclusion mean and how it is supported by the results. The authors presented the S100 system earlier (https://www.nature.com/articles/ng.3733) and so are the capabilities of doing CRISPR/Cas9 with it (https://www.cell.com/cell/fulltext/S0092-8674(17)30486-5). Here the authors present the genome assembly for S100. Are there other improvements that have been made in the system as well? If the assembly is the only improvement, then it should be made clear how the automated assembly with RagTag improves the usability of S100 in contrast to an assembly from current methods with more manual curation.

***Response:***

***We appreciate the Reviewer’s question about the novelty and potential of the S100 genotype. Indeed, the S100 genotype was first used for CRISPR-Cas9 experiments to validate the function of an inflorescence gene (LIN) in Soyk et al., 2017 (Cell) and two flowering time genes (SP and SP5G) in Soyk et al., 2017 (Nat Genet). These previous experiments suggested that the rapid flowering sp sp5g S100 genotype could be used as an efficient background for genetics and genome editing at higher throughput. However, gRNA design, off-target predictions, and indel detection by next-generation sequencing was complicated without a genome assembly. Our S100 reference assembly now allows the characterization of genome-edited plants via genome-wide approaches to quantify changes in transcription, chromatin accessibility, transcription factor binding, and more. The automation of assembly scaffolding significantly accelerated the generation of a reference assembly for this new functional system compared to manual curation.***

***Unlike in the aforementioned previous papers, we quantify in this manuscript for the first time the physiological parameters that explain the advantages of the S100 genotype compared to other widely-used tomato genotypes (M82 and Microtom). We show that generation time, plant size, seed number, and the lack of developmental abnormalities, which are important parameters for genetics experiments, make S100 an efficient experimental system for genetics. We included an extended dataset on fruiting time in a revised Fig. 1b. We also included a new dataset showing that CRISPR-Cas9 induced mutations correlated with quantitative phenotypic changes (see revised Figure 2l-p). This comprehensive quantitative data should, unlike our previous papers, demonstrate to readers specifically why and how S100 is a useful system for genetics research.***

***Text changes:***

***On page 2, we now write:***

***Previously, we used clustered regularly interspaced short palindromic repeat (CRISPR)-Cas9 genome editing to engineer mutations in the paralogous flowering repressor genes SELF PRUNING (SP) and SELF PRUNING 5G (SP5G) in S100 to induce fast flowering and compact growth [14]. We confirmed that null mutations in these genes cause rapid-cycling and compact growth without severe developmental abnormalities (Fig. 1a and Supplementary Fig. 1a-g). Furthermore, we quantified flowering and ripening time in S100, and found that the first ripe fruits mature in only ~65 days after sowing, allowing up to five generations per year compared to at most three generations for most large-fruited genotypes (Fig. 1a-d). The short generation time and compact growth habit of S100 allows both greenhouse and field growth at high density and reduced input. Notably, S100 yields ripe fruits ~3 weeks earlier than Micro-Tom and produces more seeds per fruit (Figure 1b and Supplementary Fig. 1d,f).***

***On page 4, we now write:***

***We then used multiplexed amplicon sequencing and genotyped a population of 184 T1 individuals for edits within protospacer sequences (see Methods section “Validation of CRISPR-Cas9 editing”). We identified multiple allelic combinations of j2CR, ej2CR and linCR mutations and found that higher read modification rates led to increases in inflorescence branching, supporting our previous findings that lower MADS-box gene dosage causes quantitative increases in inflorescence complexity [28](Fig. 2i). Together, these results illustrate the effectiveness of S100 as an experimental platform for CRISPR-Cas9 genome editing of individual genes and multigene families for revealing complex genotype-to-phenotype relationships.***

***On page 5, we now write:***

***In this report, we introduced a new toolset for automated genome assembly scaffolding to generate new reference genomes and elevate a rapid-cycling tomato variety to an effective experimental system for functional genomics and genome-scale editing experiments.***

Reviewer 1 comment 7:

At page 3: line 25, would not Soyk et al. Nat Gen 2016 be a more suitable citation?

***Response:***

***We agree, and cite now Soyk et al., 2017 (Nat Genet) https://doi.org/10.1038/ng.3733.***

Reviewer 1 comment 8

Page 4: line 36, typo in fig reference.

***Response:***

***We thank the reviewer for pointing out this oversight and have modified the figure reference.***

***Text changes:***

***On page 4, we now write:***

***When comparing all three genomes, we confirmed elevated rates of structural variation across broad chromosomal regions, indicating introgressions from wild relatives (Fig. 1i-k).***

Reviewer 1 comment 9

Page 5: line 6, in ap3CR it is not clear what is CR.

***Response:***

***We agree, and have modified the text and legend to improve clarity.***

***Text changes:***

***On page 4, we now write:***

***We identified multiple ap3 CRISPR-Cas9 mutant alleles (ap3CR) by Sanger sequencing and observed germline transmission in the next generation, demonstrating efficient and robust editing in S100 (Fig. 2c, d and Supplementary Fig. 3c-e).***

Reviewer 1 comment 10

Page 6: line 23, function h() is referred but I could not find it in Methods.

***Response***

***Thank you for pointing out this omission. The new methods section “RagTag “merge”” now has a description of this function, which is the scaffold graph weights from the SALSA2 algorithm.***

Reviewer 1 comment 11

Related to 4), would be better if reference to Method sections also include name of the sub-sections.

***Response:***

***Thank you for this suggestion. We agree that this would improve clarity and readability, and have implemented these changes throughout the text.***

Reviewer 1 comment 12

Supp Fig 1, do not have 'n' as mentioned in the caption.

***Response:***

***We apologize for the oversight and have indicated all replicate numbers in Supplementary Fig. 1 with an “n”.***

Reviewer 1 comment 13

The sequencing data is available on SRA, the assemblies should also be uploaded to standard public servers (NCBI , ENA etc).

***Response:***

***We thank the reviewer for this comment and we strive to make the assemblies and annotation as accessible to the community as possible. In addition to our “Pan-Solanum Genomes Project” GitHub repository (https://doi.org/10.5281/zenodo.6814693), we also uploaded the assembly and annotation files to the widely-used Solanaceae community server (solgenomics.net). Solgenomics has been the steward of the tomato reference genome for 10 years and is the primary resource for genomes in the Solanaceae community (tomato, pepper, eggplant, potato, etc).***

***Text changes:***

***On page 17, we now write:***

***Genome assemblies and annotations are available at https://github.com/pan-sol/pan-sol-data (https://doi.org/10.5281/zenodo.6814693), and the Solanaceae Genomics Network (https://solgenomics.net/ftp/genomes/M82/; https://solgenomics.net/ftp/genomes/Sweet-100/)***

Reviewer 2 comment 0

This manuscript describes the assembly of high-quality chromosome-scale genomes of two tomato cultivated accessions (M82 and Sweet-100) using their newly developed (or improved) package RagTag and the promotion of a rapid-cycling and compact growth variety (Sweet-100) as an effective system for tomato functional genomics and genome editing studies. Overall, the manuscript is well-written and the resources and computational programs developed by the authors are very useful and valuable.

***Response:***

***We appreciate the reviewer’s positive feedback about our manuscript, and the notion that both the S100 reference assembly and the RagTag software will be valuable resources for the scientific community. We also thank the reviewer for helpful suggestions to improve the clarity of our manuscript. We address all specific comments, questions, and suggestions in detail below.***

Reviewer 2 comment 1

Page 3, Line 43, "RagTag, a new toolset": Since RagTag seems to be a newer version of RaGOO, maybe change this to "RagTag, an improved version of RaGOO with several newly added functions"?

***Response:***

***We thank the reviewer for this suggestion and modified the text.***

***Text changes***

***On page 2, we now write:***

***To help overcome the limitations of current assembly scaffolding approaches and rapidly generate reference genomes for S100 and M82, we developed RagTag, an improved version of RaGOO to automate scaffolding and improve modern genome assemblies (Fig. 1e).***

Reviewer 2 comment 2

Page 3, Line 55: "[12]. [12]" -> "[12]."

***Response:***

***We apologize for the oversight and deleted the duplicated citation in the text.***

Reviewer 2 comment 3

Page 4, Line 36: "Fig. 1j,kf,g" -> "Fig. 1j,k"

***Response:***

***We thank the reviewer for pointing out this oversight and have corrected the figure reference (see Reviewer 1 comment 8).***

Reviewer 2 comment 4

Page 11, Line 30-49: I don't think this paragraph fits well to this manuscript. I would suggest to move this paragraph to the RagTag website.

***Response:***

***We thank the reviewer for the comment about our analyses of the human T2T genome assembly and respectfully disagree to remove the paragraph from the manuscript text. We conducted the benchmarking analyses on human data to evaluate the RagTag “patch” function and how automatic RagTag patches compare to the manual patches that were conducted during scaffolding of the CHM13v0.7 assembly. We found that RagTag made all 25 patches, 19 of which were identical to the manual patches, and 6 were only slightly shifted due to local repetitive sequences. RagTag placed only one false patch that was due to a misassembly in CHM13v0.7. These results show that RagTag reliably patches draft assemblies using exciting assemblies. The importance of the benchmarking analyses with human T2T data were also pointed out by Reviewer 3 (comments 6, 7, and 9). We therefore prefer to retain the paragraph in the manuscript.***

Reviewer 2 comment 5

Page 13, Line 31: Wrong citation for Winnowmap2. Ref. 23 is for Minimap2. Should be https://www.biorxiv.org/content/10.1101/2020.11.01.363887v1

***Response***

***We apologize for the oversight and now cite the correct literature. Note the peer reviewed version of the Winnowmap2 paper is now published in Nature Methods: https://www.nature.com/articles/s41592-022-01457-8***

Reviewer 2 comment 6

Page 13, Line 48 and other places: The authors mentioned using Hi-C data to help scaffold contigs of both Sweet-100 and M82. Are the data generated by the authors under this study? I couldn't find the description of Hi-C data generation. Or the data were downloaded from public depositories? Need to make this clear.

***Response***

***We thank the reviewer for this comment and acknowledge that although we mentioned the Hi-C data generation By Arima Genomics in the methods and deposited the data under the linked bioproject, the methods could have been more clear.***

***On page 13 in the methods section “Extraction of high-molecular weight DNA and sequencing”, we now write:***

***Hi-C experiments were conducted with 2 g of flash-frozen leaf tissue using the Arima high coverage Hi-C Service at Arima Genomics (San Diego, CA).***

Reviewer 2 comment 7

Page 14, Line 14-17: Why the tomato chloroplast and mitochondrion genomes from GenBank were added to final assembly? These should be independent of nuclear genome assemblies.

***Response***

***Adding these organelle genomes are useful because reads emanating from these genomes often appear (sometimes in rather high abundance) in DNA sequencing runs. For example, it is common for many RNA-seq reads to emanate from these organelle genomes. If references for these genomes are not available, then these reads can be spuriously mapped to the nuclear genome, especially for nuclear genomes that contain mitochondrial or plastid genome insertions (such as A. thaliana Columbia-0). Therefore, we think that including these genomes is useful for users, and users who wish to remove them can do so easily with standard bioinformatics software.***

***We acknowledge that there is no set convention for this. Some reference assemblies contain organellar references and some do not. However, the convention set by the T2T consortium [4] and the Vertebrate Genomes Project [5] is to include organelle genomes, and we would prefer to follow their example.***

Reviewer 2 comment 8

Page 14, Line 24: Ref. 35 should be cited for Sniffles. Remove Ref. 35 in Line 27.

***Response:***

***We apologize for the oversight and now cite the correct literature.***

Reviewer 2 comment 9

Page 15, Line 11: Change "Mercury" to "Merqury". Need to indicate what data were used for Merqury analyses. Illumina short reads or HiFi long reads…

***Response:***

***We appreciate the reviewer’s comment and modified the text to specify which data was used for the Merqury analyses.***

***Text changes:***

***We now state that “Merqury was used to calculate QV and k-mer completeness metrics using 21-mers from the HiFi data. ”***

Reviewer 3 comment 0

The manuscript is introducing RagTag as a successor to Ragoo. Ragoo in itself was highly useful already and I think RagTag is a very useful contribution.

***Response:***

***We appreciate the reviewer’s comment about our previous work that introduced the assembler RaGOO and pointing out that RagTag will likely also be widely adopted by the community. We also thank the reviewer for the comments about improving the structure of the manuscript and including additional information and data on the RagTag tool. We address all specific comments and suggestions in detail below.***

Reviewer 3 comment 1

I guess dictated by the form(?) the manuscript presents RagTag - but not in the depth it deserves - and also genome editing results. Like this I have a feeling the manuscript is neither fish nor fowl....

***Response:***

***We thank the reviewer for the comment about the manuscript format. However, we respectfully disagree that our computational (RagTag development and genome assemblies) and molecular (S100 genome editing) work do not go hand in hand in this manuscript. With this work, we aim to illustrate how advances in assembly scaffolding allow the rapid generation of reference assemblies for any given genotype of interest. Genome-editing tools can then be optimized to genetically interrogate those genotypes.***

Reviewer 3 comment 2

Considering this should be a toolpaper for RagTag:

The individual assembly statistics (N50, 70 etc) for the Sweet-100 tomato assembly should be presented and at least some dot plots between the hifi and the ONP assembly be shown maybe referenced versus the reference tomato genome.

***Response:***

***We appreciate the reviewer’s comment. For M82 and S100 assembly statistics, we point the reviewer to the main text on page 3, Figure 1h and Supplementary Table 3. Furthermore, we show synteny plots comparing M82 and S100 assemblies to the Heinz 1706 SL4.0 reference genome in Figures 1j and 1k. These are not dotplots per se, but they convey the same information. In addition to these, we have added the dotplots in Supplementary Figure 2 comparing M82 and S100 HiFi and ONT assemblies to the SL4.0 reference genome.***

Reviewer 3 comment 3

The authors might want to investigate other assemblers for the ONP data Vanrengs reported better results for the -however seemingly unmaintained - necat assembler;

***Response:***

***We thank the reviewer for this suggestion. We agree that we might observe slight differences in the results if we had used a different genome assembler for the ONT data. However, we note that the Flye assembler is an industry standard and we think that the Flye assembly results are quite good. We also believe that comparing genome assembly performance would be beyond the scope of this paper.***

Reviewer 3 comment 4

Definitely a comparison to quickmerge needs to be included as this can be used for merging ONT + hifi data as well

***Response:***

***We thank the reviewer for this comment about comparing RagTag to quickmerge. Reviewer 1 raised a similar comment about comparisons with other reconciliation tools. We address this comment in detail in the response to Reviewer 1’s comment 3.***

Reviewer 3 comment 5

The authors should best provide this test case in the github and state what should be the best strategy for Patching hifi with ONP or vice versa? The strategy is hidden in the methods section here and it makes sense as hifi is more base-correct as discussed but for maximal usability a clear guideline should be given in the documentation.

***Response***

***Thank you for this suggestion as this is a common question from users. We have added our advice to the GitHub documentation: https://github.com/malonge/RagTag/wiki/FAQ#2-which-assembly-should-be-the-target-vs-query-for-patching***

Reviewer 3 comment 6

For the human genome I think this deserves a figure in the main manuscript as well.

***Response:***

***We thank the reviewer for this suggestion. The human genome analysis serves as a nice benchmark for RagTag patch, since those patches were manually made and validated for accuracy by the T2T consortium. However, because this is a technical benchmark, we prefer to keep the same narrative and theme, specifically about model systems for plant genomics.***

Reviewer 3 comment 7

Can the error in the patched human genome be resolved by using a different ONP genome?

***Response:***

***Yes, we believe that the error was caused by a false-join misassembly in CHM13v.07. If another assembly was used for patching that did not contain this misassembly, RagTag would not have made this error. We used CHM13v.07 as it is/was the best ONT-based assembly available according to the T2T consortium.***

Reviewer 3 comment 8

Would there be any warnings in the the RagTag patch procedure so one could be aware of this problem

I am asking this question as I suspect but might be wrong that RagTag is going to be highly used for non model genomes and to investigate structural variation in human genomes where both technologies are available. Here it was easy to find such an issue (it is major and easy to spot) but what about lesser problems

***Response:***

***RagTag offers a variety of tools that can be useful for detecting misassemblies. RagTag “correct” can identify and correct some types of misassemblies, as described in the RaGOO paper, the methods of this manuscript, and the RagTag documentation. One could conceivably also use RagTag “scaffold” to quickly check how a new assembly compares to an established reference genome. The AGP file utilities can also be very useful for manual scaffolding correction. Outside of RagTag, there are many tools that can be useful for correcting misassemblies. Unlike tools that are designed for assembly correction, RagTag “patch” will trust any patch that is well supported by unique alignments. Therefore, it is very important that users use accurate input assemblies and that they validate RagTag output.***

Reviewer 3 comment 9

In the same vein maybe the authors should assembly non perfect human genome assemblies and test RagTag on these to investigate the potential performance in high throughput cancer genome analysis. The biological story could then be a nice add on story to show that RagTag is not only an interestig tool but that it has direct applications

***Response:***

***We thank the reviewer for this suggestion and we agree that such an analysis would be very interesting. However, we respectfully believe that such analysis would be beyond the scope of this work and would be better suited for a separate paper. The M82 and S100 assembly results demonstrate RagTag’s accuracy and applicability to realistic modern genome assemblies. Indeed, the CHM13 T2T assembly is not representative of most genome assemblies, but it serves as a valuable “ground-truth” for benchmarking. Relatedly, please see our response to Reviewer 1 comment 1 for an analysis of how input assembly quality impacts RagTag results for A. thaliana genome assemblies.***

**References:**

**1. Naish M, Alonge M, Wlodzimierz P, Tock AJ, Abramson BW, Schmücker A, et al. The genetic and epigenetic landscape of the centromeres. Science. 2021;374:eabi7489.**

**2. Edger PP, Poorten TJ, VanBuren R, Hardigan MA, Colle M, McKain MR, et al. Origin and evolution of the octoploid strawberry genome. Nat Genet. Nature Publishing Group; 2019;51:541–7.**

**3. Alonge M, Shumate A, Puiu D, Zimin AV, Salzberg SL. Chromosome-Scale Assembly of the Bread Wheat Genome Reveals Thousands of Additional Gene Copies. Genetics. Oxford Academic; 2020;216:599–608.**

**4. Nurk S, Koren S, Rhie A, Rautiainen M, Bzikadze AV, Mikheenko A, et al. The complete sequence of a human genome. Science. 2022;376:44–53.**

**5. Rhie A, McCarthy SA, Fedrigo O, Damas J, Formenti G, Koren S, et al. Towards complete and error-free genome assemblies of all vertebrate species. Nature. Nature Publishing Group; 2021;592:737–46.**

**Second round of review**

**Reviewer 1**

I would like to thank the authors for detailed answers and descriptions for my earlier comments. Their efforts in adding new benchmarks for comprehensive review of the 'patch' and 'merge' algorithms are highly appreciated. I find the bench-marking strategy mostly reasonable, but the new results are quite surprising, specifically Supplementary Table 1 and 2. I suspect that there might be errors while creating these tables, however, for the purpose of this review I will assume that all results are error-free and will raise my concerns accordingly.

Major comments:

1) The "patch" algorithm "fills" and "joins" contigs. However, for four of the five contig-level thaliana assemblies analysed here, the number of contigs is same after using "patch", implying that no contigs were joined (Supplementary table 1). Further, most assembly statistics are rather unaffected by patching, specially for the in-house generated assemblies. Does this mean that in most cases "patching" would have no effect on assembly quality? Are there specific conditions only under which the ragtag patch could join contigs?

2) I find the use of assembly from PRJEB37252 an efficient way to demonstrate the applications of "patch: fill". This original assembly has 23 gaps but it is not clear how many of these could be filled by ragtag? Quast results show that the number of mis-assemblies change from 38 to 32. Does this mean that 6 of the initial gaps were filled? If so, why only 6? Or were all 23 of the original gaps were filled but some of the fills resulted in misassemblies? This raises question on the accuracy of the patching.

3) The manuscript mentions that ragtag results are affected by input assembly. However, it is not clear how the assembly quality would affect path/merge. For example, in Supplementary Table 1, among the contig level assemblies, the A. thaliana assembly generated using Verkko with the highest auNG and the PRJEB23977 assembly with the lowest auNG can be considered as the best and the worst input assemblies, respectively. But both of them are mostly unaffected by ragtag patch. On the other hand, the assembly of GCA_927323615 for which the auNG is somewhere in the middle, gets improved. A more detailed description of the effects of assembly quality on the two new algorithms would significantly improve the manuscript and usability.

4) Also, though assembly metrics using QUAST are great to have, statistics for the direct applications of ragtag (number of contigs joined, number of gaps filled, accuracy/error-rate for gap-filling, length distribution of filled gaps, genomic properties of the filled gapped: homopolymeric vs tandem repeats vs centromeric, number of scaffolds corrected, number of misassemblies induced etc) would better describe the applicability.

5) I respectfully disagree with the conclusion that "merge" validates homology-based scaffolding, because the homology based scaffold itself was used as input to the merge. Such a validation would require comparison against merged assemblies generated using non-homology based scaffolds.

6) Based on the current results, the utility of "merge" as a scaffold reconciliation tool remains unclear. The application on tomato genomes resulted in the homology-based assembly for M82 and for S100, based on the QUAST results, I guess one extra edge was added in the homology based scaffold-graphs. This suggests that "merge" has limited applicability in improving scaffolding when homology-based assemblies can be generated. "Merge" could be useful when homology-based scaffolds cannot be generated or expected to be wrong (example: when no reference genome is present or the reference genome is diverged compared to focal genome). Tests with "merge" in such scenarios could provide better insights to its utility.

7) The "merge" results for the thaliana genomes (Supplementary Table 2, ST2) are unfortunately confusing. The input is same as Supplementary Table 1 (ST1), even though ST1 shows results for "patch" that requires contigs as input whereas ST2 shows results for "merge" that requires scaffolds as input.
The A. thaliana genomes were scaffolded using homology with multiple reference genomes and then merged (page 12, line 51). So, I assume that, for each merge run there would be multiple input assemblies. However, ST2 has only single input.
The HIFIASM.L1.output and HIFIASM.L3.output are exactly same. Futher they have more contigs then the input file. How could this be possible? Some description for this would be very helpful.

8) Do the L1/L3 in HiFiasm.L1/HiFiasm.L3 refer to the purging level? If so, then what is the reasoning behind using these values when the developers of Hifiasm recommend using -l0 for homozygous genomes (https://hifiasm.readthedocs.io/en/latest/faq.html?highlight=homozygous#are-inbred-homozygous-genomes-supported)?

Minor comments:

1) Typically assembly accession number is used to refer an assembly rather BioProject number.

2) I cannot find the zoomed-in contacts maps in the new Figure S3.

3) For Supplementary Table 4/5 legends are incorrect.

4) I think, it would be better if the CR in ap3CR is defined at its first occurence (page 4: line33).

**Reviewer 2**

The authors have addressed all my concerns.

**Authors Response**

**Point-by-point responses to the reviewers’ comments:**

Response to the reviewer’s comments:

Reviewer #1: I would like to thank the authors for detailed answers and descriptions for my earlier comments. Their efforts in adding new benchmarks for comprehensive review of the 'patch' and 'merge' algorithms are highly appreciated. I find the bench-marking strategy mostly reasonable, but the new results are quite surprising, specifically Supplementary Table 1 and 2. I suspect that there might be errors while creating these tables, however, for the purpose of this review I will assume that all results are error-free and will raise my concerns accordingly.

***Response: We thank the reviewer for raising these points. We agree that the initial benchmarking analysis was unclear and incomplete. Using your suggestions, we have completely redone these analyses using new assemblies and new quality metrics as we describe in more detail below.

Aside from providing standard assembly statistics, we also now assess scaffolding accuracy with dotplots provided in Supplementary Fig. 2-10. For patching, we assessed patch accuracy by aligning the patches back to the Col-CEN reference and summarizing the coverage and identity of the corresponding alignments.

For all analyses, we screened input contigs using the same method described for the tomato assemblies. Because all assemblies represent the same Col-0 haplotype, for the patch benchmarking, we also ran RagTag “correct” on the input assemblies to improve accuracy.***


Major comments:

1) The "patch" algorithm "fills" and "joins" contigs. However, for four of the five contig-level thaliana assemblies analysed here, the number of contigs is same after using "patch", implying that no contigs were joined (Supplementary table 1). Further, most assembly statistics are rather unaffected by patching, specially for the in-house generated assemblies. Does this mean that in most cases "patching" would have no effect on assembly quality? Are there specific conditions only under which the ragtag patch could join contigs?

***Response: After analyzing the initial results, we realized that the centromeres in the Col-CEN reference assembly were inhibiting patching. RagTag looks for potential patches by identifying unique alignments between one “query” assembly contig and multiple “target” assembly contigs. Since each query contig contained a complete centromere sequence, there was a break in the chain of unique alignments at the centromere, and that caused no patching for the entire chromosome.

To overcome this, we broke the Col-CEN assembly into arms and used this new assembly as the “query” assembly for patching. This means that patching could, at most, reconstitute the chromosome arms. However, we now see much more patching when using this new reference.

We recognize that this may not be straightforward for users who are using complete reference assemblies. Accordingly, we have added documentation (https://github.com/malonge/RagTag/wiki/patch#using-complete-query-assemblies) to the GitHub repo outlining best practices for how to use highly complete assemblies like Col-CEN or the human T2T reference.***

2) I find the use of assembly from PRJEB37252 an efficient way to demonstrate the applications of "patch: fill". This original assembly has 23 gaps but it is not clear how many of these could be filled by ragtag? Quast results show that the number of mis-assemblies change from 38 to 32. Does this mean that 6 of the initial gaps were filled? If so, why only 6? Or were all 23 of the original gaps were filled but some of the fills resulted in misassemblies? This raises question on the accuracy of the patching.

***Response: RagTag patch always breaks input assemblies at gaps to produce contigs. Therefore, there is no functional difference between using scaffolds or contigs as input into RagTag. To simplify the interpretation of the analysis we always broke scaffolds into their contigs (this only applied to GCA_902825305, as you point out). In our new analysis, RagTag ultimately made 11 patches for this contig-level assembly.***

3) The manuscript mentions that ragtag results are affected by input assembly. However, it is not clear how the assembly quality would affect path/merge. For example, in Supplementary Table 1, among the contig level assemblies, the A. thaliana assembly generated using Verkko with the highest auNG and the PRJEB23977 assembly with the lowest auNG can be considered as the best and the worst input assemblies, respectively. But both of them are mostly unaffected by ragtag patch. On the other hand, the assembly of GCA_927323615 for which the auNG is somewhere in the middle, gets improved. A more detailed description of the effects of assembly quality on the two new algorithms would significantly improve the manuscript and usability.

***Response: We agree that our analyses were not fully clear about how input assembly quality affects RagTag results. We believe the new analysis, which we present in Supplementary Table 1, is much more consistent with expectations: less contiguous assemblies are patched more.***

4) Also, though assembly metrics using QUAST are great to have, statistics for the direct applications of ragtag (number of contigs joined, number of gaps filled, accuracy/error-rate for gap-filling, length distribution of filled gaps, genomic properties of the filled gapped: homopolymeric vs tandem repeats vs centromeric, number of scaffolds corrected, number of misassemblies induced etc) would better describe the applicability.

***Response: We thank the reviewer for recommending these helpful statistics, which are now included in Supplementary Table 1. Upon reflection, we found that the QUAST stats were not very useful, in part because the Col-CEN reference is so complete and QUAST is not robust to large repeats such as centromeres and also because the input and output assemblies are so similar. We decided to more directly measure scaffolding accuracy with dotplots in a new Supplementary Fig. 2. We also specifically measured patch accuracy in the Arabidopsis assemblies by cutting out the patch and flanking sequences and aligning the patches back to the Col-CEN reference assembly. In addition we quantified the number of simple repeats, satellite repeats, and transposable elements in the patched sequences.***

5) I respectfully disagree with the conclusion that "merge" validates homology-based scaffolding, because the homology based scaffold itself was used as input to the merge. Such a validation would require comparison against merged assemblies generated using non-homology based scaffolds.

***Response: We apologize for the misunderstanding regarding this claim. We were referencing how, for the tomato assemblies, we used RagTag to merge homology-based scaffolds with Hi-C based “de novo” scaffolds. For this analysis, we re-weighted the underlying scaffold graph data structure using the same Hi-C “best buddy scaling” method developed by SALSA2. Consequently, the output contigs were identical to the input homology based scaffolds, which means that the “de novo” scaffolds did not contradict the homology-based scaffolds. By using scaffold graphs to compare homology-based and Hi-C based joins, we can say that this serves as an automatic validation of the homology-based scaffolds. In other words, all of the joins that SALSA2 would have made were also made in the homology-based scaffolds.***

6) Based on the current results, the utility of "merge" as a scaffold reconciliation tool remains unclear. The application on tomato genomes resulted in the homology-based assembly for M82 and for S100, based on the QUAST results, I guess one extra edge was added in the homology based scaffold-graphs. This suggests that "merge" has limited applicability in improving scaffolding when homology-based assemblies can be generated. "Merge" could be useful when homology-based scaffolds cannot be generated or expected to be wrong (example: when no reference genome is present or the reference genome is diverged compared to focal genome). Tests with "merge" in such scenarios could provide better insights to its utility.

***Response: We agree with the reviewer that RagTag “merge” can be useful in various situations depending on what maps are available for the particular project. We highlight the combination of homology maps (reference genomes) and Hi-C maps because these are highly relevant and effective maps for modern genome assemblies.***

***As discussed in the previous comment, we believe that the “merge” utility was a useful validation of the M82 homology-based scaffolds, even if it did not yield any additional scaffolding. As for S100, even though only a few additional joins were made, this potentially saved us hours of manual and tedious scaffolding to join the sequences that were joined by RagTag “merge”. A central theme of this manuscript is that, though genome assembly is very advanced, finishing assemblies (including scaffolding and polishing) is usually done manually, and even small automatic improvements can be very valuable towards the end of assembly pipelines. Outside of the work we present here, this manual curation step was needed for the human T2T assembly, the Arabidopsis Col-CEN assembly, and many more. RagTag is a suite of tools that all play different roles and can provide varying utility depending on the genome assembly project at hand. We feel that, based on the results presented in the manuscript, our own experiences generally, and the popularity of the software, all of RagTag tools are valuable contributions to the genome assembly community.***

7) The "merge" results for the thaliana genomes (Supplementary Table 2, ST2) are unfortunately confusing. The input is same as Supplementary Table 1 (ST1), even though ST1 shows results for "patch" that requires contigs as input whereas ST2 shows results for "merge" that requires scaffolds as input.
The A. thaliana genomes were scaffolded using homology with multiple reference genomes and then merged (page 12, line 51). So, I assume that, for each merge run there would be multiple input assemblies. However, ST2 has only single input.
The HIFIASM.L1.output and HIFIASM.L3.output are exactly same. Futher they have more contigs then the input file. How could this be possible? Some description for this would be very helpful.

***Response: We thank the reviewer for pointing out this missing information. We now include stats for all the assemblies used for merging, including the intermediate scaffolded assemblies, in a revised Supplementary Table 2, and present dotplots for input and output assemblies in new Supplementary Figures 3-10. The additional dotplots capture the fact that the merged assembly quality is highly dependent on input assembly quality (as expected) and that merging is done in a conservative manner to reduce mis-scaffolding (also as expected).***

8) Do the L1/L3 in HiFiasm.L1/HiFiasm.L3 refer to the purging level? If so, then what is the reasoning behind using these values when the developers of Hifiasm recommend using -l0 for homozygous genomes (https://hifiasm.readthedocs.io/en/latest/faq.html?highlight=homozygous#are-inbred-homozygous-genomes-supported)?

***Response: We thank the reviewer for this comment. We now only use “-l0” for the Hifiasm assemblies.***


Minor comments:

1) Typically assembly accession number is used to refer an assembly rather BioProject number.

***Response: Thank you for pointing this out — we now refer to the assemblies using their Genbank accession code.***

2) I cannot find the zoomed-in contacts maps in the new Figure S3.

***Response: We apologize for the misunderstanding, but the zoomed in contact maps are provided to you personally but not added to the manuscript for publication. As we stated before, we think that the loci that you point out are correctly assembled/scaffolded, but there are signatures of 3D structure that cause the patterns that you mentioned. The zoomed in contact info can be found here: https://drive.google.com/drive/folders/1yTBe_YbMuTR6CmaogmEKWAEaIKuxV_Sb?usp=sharing

If you and/or the editor feels it is necessary we would be happy to include these in the supplementary results as well.***

3) For Supplementary Table 4/5 legends are incorrect.

***Response: We apologize for the oversight and corrected the legends accordingly.***

4) I think, it would be better if the CR in ap3CR is defined at its first occurence (page 4: line33).

***Response: We agree and made the text changes to now introduce the ap3CR abbreviation at its first occurrence.***
